# Does It Matter Whether You or Your Brain Did It? An Empirical Investigation of the Influence of the Double Subject Fallacy on Moral Responsibility Judgments

**DOI:** 10.3389/fpsyg.2019.00950

**Published:** 2019-04-30

**Authors:** Uri Maoz, Kellienne R. Sita, Jeroen J. A. van Boxtel, Liad Mudrik

**Affiliations:** ^1^Department of Psychology, Crean College of Health and Behavioral Science, Chapman University, Orange, CA, United States; ^2^Schmid College of Science and Technology, Chapman University, Orange, CA, United States; ^3^Institute for Interdisciplinary Brain and Behavioral Sciences, Chapman University, Orange, CA, United States; ^4^Department of Psychology, University of California, Los Angeles, Los Angeles, CA, United States; ^5^Division of Humanities and Social Science, California Institute of Technology, Pasadena, CA, United States; ^6^School of Psychology, Faculty of Health, University of Canberra, Canberra, ACT, Australia; ^7^School of Psychological Sciences, Faculty of Medicine, Nursing and Health Sciences, Monash University, Clayton, VIC, Australia; ^8^Monash Institute of Cognitive and Clinical Neurosciences, Monash University, Clayton, VIC, Australia; ^9^Sagol School of Neuroscience, Tel Aviv University, Tel Aviv, Israel; ^10^School of Psychological Sciences, Tel Aviv University, Tel Aviv, Israel

**Keywords:** closet dualism, “my brain made me do it”, moral responsibility, conceptual confusions in neuroscience, moral scenarios, Double Subject Fallacy

## Abstract

Despite progress in cognitive neuroscience, we are still far from understanding the relations between the brain and the conscious self. We previously suggested that some neuroscientific texts that attempt to clarify these relations may in fact make them more difficult to understand. Such texts—ranging from popular science to high-impact scientific publications—position the brain and the conscious self as two independent, interacting subjects, capable of possessing opposite psychological states. We termed such writing ‘Double Subject Fallacy’ (DSF). We further suggested that such DSF language, besides being conceptually confusing and reflecting dualistic intuitions, might affect people’s conceptions of moral responsibility, lessening the perception of guilt over actions. Here, we empirically investigated this proposition with a series of three experiments (pilot and two preregistered replications). Subjects were presented with moral scenarios where the defendant was either (1) clearly guilty, (2) ambiguous, or (3) clearly innocent while the accompanying neuroscientific evidence about the defendant was presented using DSF or non-DSF language. Subjects were instructed to rate the defendant’s guilt in all experiments. Subjects rated the defendant in the clearly guilty scenario as guiltier than in the two other scenarios and the defendant in the ambiguously described scenario as guiltier than in the innocent scenario, as expected. In Experiment 1 (*N* = 609), an effect was further found for DSF language in the expected direction: subjects rated the defendant less guilty when the neuroscientific evidence was described using DSF language, across all levels of culpability. However, this effect did not replicate in Experiment 2 (*N* = 1794), which focused on different moral scenario, nor in Experiment 3 (*N* = 1810), which was an exact replication of Experiment 1. Bayesian analyses yielded strong evidence against the existence of an effect of DSF language on the perception of guilt. Our results thus challenge the claim that DSF language affects subjects’ moral judgments. They further demonstrate the importance of good scientific practice, including preregistration and—most critically—replication, to avoid reaching erroneous conclusions based on false-positive results.

## Introduction

The neuroscientific study of voluntary action sparked an ongoing debate, both inside and outside of academia, about the intricate relations between the conscious self and the brain. How is our conscious experience of deciding related to the neural processes that mediate this decision? Does consciousness play a causal role in the processes that lead to action? And, if it does not, might our common notions of free will be nothing more than an illusion (Libet, 1985; Wegner and Wheatley, 1999; Wegner, 2002; Caruso, 2013)?

These discussions and debates were sometimes accompanied by various conceptual confusions. As science has been providing more insight into the human brain, scholars and the lay public began shifting the way they describe the brain and its relations to the self, not always appropriately. In popular science books and debates, which commonly lay the foundation on which lay perceptions of neuroscience are formed, the brain is often described as separate from the self, and—critically—as capable of interacting with the self while possessing opposing psychological states to those held by the self (Damasio, 1994; LeDoux, 2003; Gazzaniga, 2006; Frith, 2007; Rizzolatti and Sinigaglia, 2008). But such writing is not limited to popular science. It can often, and quite strikingly, be found in scientific papers published in leading journals (Libet et al., 1983; Keysers and Perrett, 2002; Pearson and Clifford, 2005; Cerf et al., 2010; Simon and Gogotsi, 2010). (For a full discussion, see Mudrik and Maoz, 2014.) Echoes of this type of writing can also be found in the increasing use of neuroscientific evidence in courtrooms (Garland and Glimcher, 2006; Chandler, 2016; Meixner, 2016), typically as part of the “my brain made me do it” defense (Morse, 2004; Gazzaniga, 2006; Sternberg, 2010; Maoz and Yaffe, 2015).

We previously defined such writing, which separates the brain from the conscious self and ascribes divergent psychological states to them, as the “Double Subject Fallacy” (DSF; Mudrik and Maoz, 2014). These psychological states can be more than just divergent and even oppose one another (e.g., “the brain knows our decisions before we do,” Gazzaniga, 2006). This type of writing implies that there are two intentional subjects, the person (or self) and the person’s brain, capable of interacting with one another and having different mental states, like not knowing and knowing, respectively. And so, both the self and the brain are assigned with psychological predicates and intentional states, as if these are two different subjects. We claim (also following the mereological fallacy; Bennett and Hacker, 2005) that the DSF is a form of conceptual confusion that stems from remnants of Cartesian dualism in modern science, despite scientists’ repeated claims to hold materialistic views. In this new “closet-dualism,” the mind-body dichotomy is replaced by a self-brain dualism.

It is noteworthy that it is generally rather easy to eliminate DSF language. For example, “…it seems the brain knows our decisions before we do.” (Gazzaniga, 2006, p. 45) could be rephrased as “…it seems that decision processes are carried out unconsciously before we have conscious access to them.” It is perhaps longer, but certainly more precise. Similarly, “Our brain doesn’t tell us everything it knows. And sometimes it goes further and actively misleads us.” (Frith, 2007, p. 47) could be rewritten as “We don’t have conscious access to all the information processed by our brains. And sometimes our conscious experience is further actively misleading.”

In addition, we suggested that, rather than being cute (perhaps humoristic) shorthand aiming to facilitate the understanding of complex scientific texts, the DSF does the opposite (Mudrik and Maoz, 2014). It adds unnecessary complications to the already complex problem of mind–brain relations. We further wondered whether it might bear practical implications for the judicial system, to the extent that it lays the foundations for the abovementioned “my brain made me do it” defense.

Indeed, decision making, inside and outside of the judicial system, is held to be susceptible to (unconscious) influences of irrelevant factors and preconceptions (Houses of Parliament, 2015), which may include the DSF. For example, numerous studies have focused on the effects of physical, demographic, or personality characteristics of the perpetrator, victim or the observer on blame or responsibility attributions (for review of findings, see Alicke, 2000). A prominent issue in this literature is race (e.g., Mazzella and Feingold, 1994; Sommers and Ellsworth, 2000; Mitchell et al., 2005, alongside physical attractiveness or socioeconomical status; see again Mazzella and Feingold, 1994, as well as Devine and Caughlin, 2014). Other factors, like negative pre-trial publicity (Steblay et al., 1999, or stereotypical beliefs about rape victims, for example, also influence decisions in case simulations (Ellison and Munro, 2008). In addition, a large study on the death penalty in Nazi Germany showed that the death penalty was more likely to be given during times when Nazi Germany suffered large numbers of battle deaths (Geerling et al., 2017). Taken together, this body of literature suggests that the assignment of guilt is often influenced by irrelevant factors.

Here, we set out to examine if DSF language may play a similar role in affecting guilt judgment in the judicial realm. In three studies, we investigated whether people assign less blame or guilt to defendants when their actions in moral scenarios are portrayed in textual vignettes using DSF language. We hypothesize that subjects would be more lenient with a defendant when her actions are described as “her brain made her do X” as opposed to “her emotions made her do X,” for example. Such a finding would demonstrate that DSF language—which is in line with implicit dualistic notions of the brain as separated from the conscious self—affects people’s judgment of guilt in moral scenarios. Therefore, people may assign less moral responsibility to agents in moral scenarios when DSF language is used. This is, perhaps, because with DSF language the brain would be viewed as an ‘external’ constraint that is put on the conscious self. Such external constraints typically lead to judging an action as less free, and—accordingly—lessening the moral responsibility associated with it (Howard-Snyder and Jordan, 1996; Nahmias et al., 2006).

Previous studies have shown that generally using neuroscientific language—irrespective of the DSF—affects subjects’ judgments. Provided with neuroscientific evidence, mock jurors list significantly more mitigating factors, or reasons to reduce the severity of a charge or the length of jail time, in their sentencing rationalizations (Aspinwall et al., 2012). They are also less likely to sentence defendants to life in prison (Greene and Cahill, 2012; see also Gurley and Marcus, 2008) when any neuroscience-based evidence was presented by the defendant. Accordingly, it has been suggested that advances in neuroscience shape people’s perspective on moral responsibility, causing them to value neuroscientific over alternative forms of evidence (Greene and Cohen, 2004). Therefore, it may very well be that using DSF language further increases these effects, thereby lessening the moral responsibility attributed to defendants.

To isolate the effect of the DSF from the more general one of neuroscientific evidence, we presented subjects with the same moral scenario that included evidence regarding the neurological state of the defendant, while simultaneously presenting neuroscientific evidence either using DSF language or using neutral, non-DSF language. Perceived moral responsibility was assessed by asking subjects to determine the guilt of the defendant and, in the first experiment, to further recommend jail sentences. We expected the implied dualism, inherent in DSF language, to make subjects judge the defendants as less morally responsible, or guilty, for their actions compared to when non-DSF language was used.

## Experiment 1

The first experiment was exploratory, aimed to assess the possible effect of DSF language on judgments of moral responsibility. It was a 3 × 2, between-subjects design, with culpability and DSF language as independent variables, and attributed guilt as a dependent variable. One moral scenario with varied facts about the case represented different levels of culpability: the defendant was either (1) clearly guilty, (2) ambiguous, or (3) clearly innocent. The ambiguous scenario described an inconclusive situation, in which the defendant was neither clearly guilty nor innocent. Each level of culpability was also presented using either (1) DSF language or (2) non-DSF, neutral language. We first expected to find differential judgments of guilt based on culpability, which served as a manipulation check. But focus was on the comparison between DSF and non-DSF language, where we hypothesized that the former would lead to more lenient judgments of guilt than the latter.

### Methods

#### Subjects

A sample of 609 subjects (238 females; aged 18–75, mean age = 32.25) was recruited using Amazon Mechanical Turk (MTurk). (We predefined the sample to include 600 subjects but ended up with 609 subjects due to the MTurk’s data collection procedure.) This sample only included subjects who provided a Worker ID that was identical to their MTurk Worker ID and who additionally provided a valid completion code that matched the code assigned to them at survey completion (see below). This, and all other experiments reported in this study, were approved through the UCLA Institutional Review Board (IRB). In compliance with this approval, subjects were compensated 25 cents upon completion of the experiment.

#### Design, Apparatus, Stimuli, and Procedure

Culpability was manipulated to include three levels (guilty, unclear, innocent). A pilot experiment suggested that the three levels of culpability reliably led subjects to attribute three corresponding levels of attributed guilt to the defendants for the three vignettes. The case was either presented in DSF language, or in non-DSF language. The guilt attributed to the defendants was operationalized as subjects’ ratings of the defendant’s intent to kill the victim. Additionally, subjects recommended jail sentence, in months.

The moral scenario and corresponding questionnaires were presented using the Qualtrics online survey tool and accessed through an MTurk HIT. Unique 8-digit verification codes were distributed on Qualtrics to be submitted on MTurk to verify valid completion of the experiment.

The moral scenario involved bodily harm to the victim and was presented alongside an overview of the necessary criteria for an individual to be found guilty of assault with intent to kill (Table 1). Then, subjects were provided with three facts about the case and were informed that both the defense and the prosecution have agreed upon these facts. The first fact was consistent across all conditions, and objectively stated the nature of the event in question (Table 1, base scenario). The second varied, according to the culpability condition, to reflect guilt, ambiguity, or innocence of the defendant (Table 1, culpability condition). The third referred to the neuroscientific evidence, and either used DSF language or non-DSF language (Table 1, DSF condition). The syntax and length of the descriptions were held constant between DSF/non-DSF conditions to maintain the consistency of the language used.

**Table 1:** Experiments 1 and 3 vignette manipulation by condition.

	Base scenario		
	Jim is being charged with vehicular assault with intent to kill. To be found guilty, it must be demonstrated that (A) he struck another person with a vehicle and that (B) he intended to kill them. The established facts of the case, on which the defense and prosecution agree, are:		
	1. Bob was struck by Jim’s car while Jim was behind the wheel. Bob was badly hurt.		
**Culpability condition**	**Guilty**	**Unclear**	**Innocent**
Second fact manipulated	2. Just before Bob was struck, Jim yelled, “I’m going to kill you, you bastard!”	2. Just before Bob was struck, Jim accelerated through a red light.	2. Just before Bob was struck, Jim slammed on the brakes and yelled, “Oh no! I’m going to hit him!”
**DSF condition**	**DSF used**		**No DSF used**	
Third fact manipulated	3. An area of Jim’s brain commonly associated with aggression is more active than the average. So Jim’s brain makes him have aggressive feelings more often than most people. Jim often struggles with his brain and tries to prevent these aggressive feelings from being expressed.		3. An area of Jim’s brain commonly associated with aggression is more active than the average. So Jim has aggressive feelings more often than most people. Jim often struggles with himself and tries to prevent these aggressive feelings from being expressed.	

The vignette was followed by a field containing two 7-point, discrete Likert scales. First, subjects were asked to rate the extent to which they agree or disagree with the sentence “Jim is guilty of the charge of vehicular assault with intent to kill.” There, 1 corresponded to “strongly disagree” and 7 to “strongly agree.” Then, they were asked to recommend a jail sentence, in months, from 0 months to a life sentence (given in bins: 0 months; 1–12 months; 13–60 months; 61–120 months; 121–240 months; 241–420 months; and life sentence).

Finally, subjects were asked to provide demographic information: age, gender, and highest level of education completed. Questions for all blocks were forced response, and subjects were asked to enter the information to proceed to the next stage of the survey. After all blocks were completed, subjects were provided with a randomized, 8-digit, unique code to submit to MTurk. The blocks had no individual time limit; however, subjects had to complete the experiment within 8 min.

#### Analysis

A classical, null-hypothesis significance testing (NHST) two-way analysis of variance (ANOVA) was run, with culpability and DSF as factors. All analyses were complemented by a Bayes-factor analysis to assess the conclusiveness of the results. Bayes factors (BF), defined as the ratio of the probability of observing the data given H_0_ and the probability of observing the data given H_1_, were calculated using JASP (JASP Team, 2018) with default settings. That is, for factorial analyses, we used the ANOVA BF with Cauchy prior width of *r* = 0.5 for fixed effects and *r* = 1 for random effects. To compute BFs for the main effects and interactions, we compared a reduced model in which the effect of interest was not included with the full model, which includes all main effects and interactions. To assess the strength of the results, we adopted the convention that a BF < 0.01 implies extreme evidence for lack of an effect, 0.01 < BF < 0.1 implies strong evidence for lack of an effect 0.1 < BF < 0.33 provides moderate evidence for lack of an effect, 0.33 < BF < 3 suggests inconclusive data, or anecdotal evidence for lack or presence of an effect, 3 < BF < 10 denotes moderate evidence for the presence of an effect, BF > 10 implies strong evidence, and BF > 100 suggests extreme evidence for the presence of an effect (Lee and Wagenmakers, 2014). The same analysis scheme was used for all three experiments.

### Results

**Figure 1:**
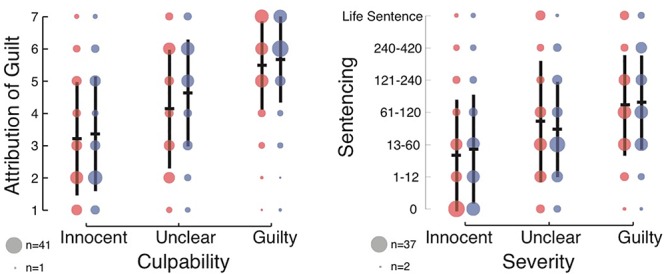
Effect of culpability (guilty, ambiguous, and innocent) and DSF language (red for DSF and blue for non-DSF) on subjects’ attribution of defendant guilt (left), and on recommended jail sentences (right). In black, central horizontal bars depict the mean ratings given by the subjects, and vertical lines reflect the standard deviation. The radii of the circles represent the number of responses for each level in the 7-point Likert scale.

As expected, the ANOVA revealed a main effect of culpability [*F*(2,603) = 98.65, *p* < 0.001, $\eta_{p}^{2}$ = 0.247, BF_10_ = 2.1 × 10^34^; see Figure 1 (left) for the full results]. Subjects assigned higher guilt to the defendant in the Guilty condition (*M* = 5.58, *SD* = 1.36), followed by the Unclear condition (*M* = 4.39, *SD* = 1.77) and the Innocent condition (*M* = 3.29, *SD* = 1.76). This validated the culpability manipulation, and provided evidence that subjects were following instructions and meaningfully attending to the text.

**Figure 2:**
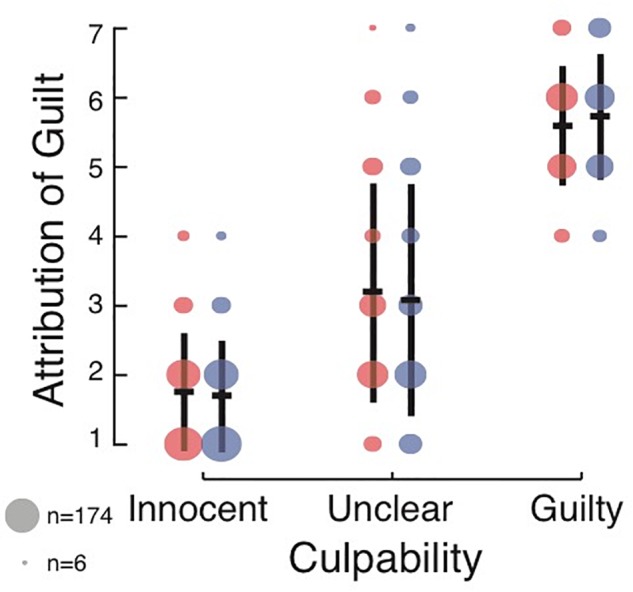
Effect of culpability and DSF language on attribution of guilt for intent to kill in Experiment 2. The annotations are the same as those used in Figure 1.

Importantly, a main effect of DSF was also found, though the Bayesian analysis suggested inconclusive or anecdotal evidence [*F*(1,603) = 4.43, *p* = 0.036, $\eta_{p}^{2}$ = 0.007, BF_10_ = 0.47]. Subjects assigned lower guilt to the defendant in the DSF condition (*M* = 4.28, *SD* = 1.91), than in the non-DSF condition (*M* = 4.56, *SD* = 1.85). No interaction was found between the factors [*F*(2,603) = 0.711, *p* = 0.49, $\eta_{p}^{2}$ = 0.002; BF_10_ = 0.065].

For the recommended jail sentences, the ANOVAs revealed a main effect of culpability [*F*(2,603) = 42.32, *p* < 0.001, $\eta_{p}^{2}$ = 0.123, BF_10_ = 8.8 × 10^14^; see Figure 1 (right) for the full results]. Subjects recommended longer sentences to the defendant in the Guilty condition, followed by the Unclear condition, and finally the Innocent condition. This further validated the culpability manipulation, and provided evidence that subjects were following instructions and meaningfully attending to the text. However, there was no main effect of DSF [*F*(1,603) = 0.01, *p* = 0.92, $\eta_{p}^{2}$ < 0.001, BF_10_ = 0.091], and subjects recommended similar sentences in the DSF and non-DSF conditions. No interaction was found between the factors [*F*(2,603) = 1.02, *p* = 0.36, $\eta_{p}^{2}$ = 0.003; BF_10_ = 0.088].

### Discussion

Experiment 1 provided first evidence for a possible effect of DSF language on the way people perceive moral responsibility: subjects who were presented with neuroscientific evidence described in DSF language were more lenient in assigning defendant guilt. This implies that DSF language might affect judgments of moral responsibility, making people assign less responsibility to others if “their brain made them do it” (Mudrik and Maoz, 2014). However, the effect we found was notably weak, and inconclusive when using Bayesian analysis. Further, we found no effect of DSF language on recommended jail sentences, and there Bayesian analysis supported the absence of an effect. There are at least two factors that could contribute to this. First, it is well known that there is large disparity for sentencing for very similar offenses (e.g., Austin and Williams, 1977). Second, subjects might have found the presentation of sentences in months confusing.

We therefore decided to continue and test the effect of DSF language specifically on guilt for another moral scenario. Finding the DSF effect on perceived guilt in a different moral scenario would provide evidence that the DSF effect is real and generalizes to other scenarios. As there was no reliable effect of the DSF on sentencing, we decided to examine the effect of DSF language only on moral responsibility in the follow-up experiment.

## Experiment 2

The main goal of Experiment 2 was to replicate the effect found in Experiment 1, though on another moral scenario. In other words, we aimed to assess both the reproducibility and the generalizability of the effect. The current vignette again involved bodily harm, yet by other means (throwing a heavy object instead of vehicular assault; see Table 2). Given the results of Experiment 1, we expected to find both effects of culpability, validating the manipulation and confirming that subjects were following instructions, and—critically—an effect of DSF language. The entire experiment—including sample size, methods, hypotheses and analysis plan—was pre-registered in the Open Science Framework (OSF^1^).

**Table 2:** Experiment 2 vignette manipulation by condition.

	Base scenario		
	Bob is being charged with battery with intent to kill. To be found guilty, it must be demonstrated that (A) he struck another person with an object and that (B) he intended to kill them. The established facts of the case, on which the defense and prosecution agree, are:		
	1. Jim was struck by Bob’s flower pot falling from the third story window. Jim was badly hurt.		
**Culpability condition**	**Guilty**	**Unclear**	**Innocent**
Second fact manipulated	2. Just before Jim was struck, Bob yelled at Jim, “I’m going to kill you, you bastard!’	2. Just before Jim was struck, Bob knocked the flower pot off of the window ledge.	2. Just before Jim was struck, Bob tried to catch the falling flower pot and screamed, “Oh no! Look out!”
**DSF condition**	**DSF used**	**No DSF used**	
Third fact manipulated	3. An area of Bob’s brain commonly associated with aggression is more active than the average. So, Bob’s brain makes him have aggressive feelings more often than most people. Bob often struggles with his brain and tries to prevent these aggressive feelings from being expressed.	3. An area of Bob’s brain, commonly associated with aggression, is more active than the average. So, Bob has aggressive feelings more often than most people. Bob often struggles with himself and tries to prevent these aggressive feelings from being expressed.	

### Methods

#### Subjects

Due to the small effect size in Experiment 1, we wanted to ensure adequate power to detect an effect in Experiment 2. A power analysis based on that effect size of Experiment 1 suggested that to reach a power of 0.95 (with alpha = 0.05) we would need 1,794 subjects^2^. We collected subjects continuously until we reached this pre-defined sample size after applying our exclusion criteria (see below).

The experiment was again run using MTurk and Qualtrics, open only to subjects who had not participated in Experiment 1. We further pre-defined exclusion criteria, based on the results of Experiment 1, for subjects who: (a) rated the defendant in the Guilty condition as a 1–3 (i.e., relatively not guilty) on the 7-point Likert scale for guilt; or (b) rated the defendant in the Innocent condition as a 5–7 on the 7-point Likert scale for intent to kill (i.e., relatively guilty), both suggesting they did not understand the task; (c) indicated with a high rating (5–7) that they regret their decision, or would like to change their answers; (d) indicated with a low rating (1–3) of confidence in their answers or that they did not feel responsible for their decision (1–3); or (e) indicated a high level of familiarity (5–7) with legal information by having taken a secondary education course on the topic or knowing the definition of the term “mens rea,” as subjects with legal knowledge cannot be regarded as lay subjects for the purpose of this experiment. As in Experiment 1, participants in this experiment also needed to provide a Worker ID that was the same as their actual MTurk Worker ID, and to provide a valid completion code that matched the code assigned at survey completion.

We collected data from 6,537 subjects. The majority of those subjects met the pre-defined exclusion criteria (which, in retrospect, were too strict; see Experiment 3): 553 (8.5%) of the collected subjects rated the defendant low in guilt in the Guilty condition (criterion a); 315 (4.8%) rated the defendant high in guilt in the innocent condition (b); 853 (13.0%) indicated high regret or wanted to change their decision (c); 491 (7.5%) indicated low confidence in their response (d); 2581 (39.5%) indicated they did not feel responsible for their response (d); 2129 (32.6%) indicated a high level of familiarity with legal information. There was some overlap in subject exclusion, yielding a final sample of 1,897 individuals (1,009 females, aged 18–82 years old; average age = 35.89 years old ± STD 11.91 years).

#### Design, Apparatus, Stimuli, and Procedure

All the above were identical to those used in Experiment 1, besides the following changes (compare Tables 1, 2): (a) the charge was changed from vehicular assault to battery, (b) the names of the defendant and the victim were swapped, (c) the first fact of the case was changed to “Jim was struck by Bob’s flower pot falling from the third story window. Jim was badly hurt,” and (d) the second fact was changed accordingly (Table 2, culpability condition).

Following the moral scenario, the same 7-point Likert scale was used as in Experiment 1, measuring perceived guilt. An ‘Additional Measures’ block was then presented, and subjects were asked to rate (a) if they were confident in their decision, (b) if they feel responsible for the outcome of the decision, (c) if they regret their decision, and (d) if they would like to change their decision. Then, subjects were asked to report their dualistic intuitions: (a) if they believe that an individual and their brain are separate entities, (b) if they believe that an individual and their mind are separate entities, and (c) if they believe that an individual’s mind and brain are separate entities. All the above ratings were given on 7-point Likert scales ranging from 1 – “no” to 7 – “yes.” Given the additional measures taken, time limits for completing the entire survey was now set to 10 min.

### Results

A two-way ANOVA with the factors culpability and DSF showed a strong main effect of culpability [*F*(2,1891) = 1634.38, *p* < 0.001, $\eta_{p}^{2}$ = 0.63; BF_10_ = 4.64 × 10^408^], such that subjects had the highest guilt ratings in the Guilty condition (*M* = 5.66, *SD* = 1.89), followed by the Unclear condition (*M* = 3.13, *SD* = 1.63) and then the Innocent condition (*M* = 1.72, *SD* = 0.83). The effect of DSF language was not significant and negligible in terms of effect size [*F*(1,1891) = 0.040, *p* = 0.84, $\eta_{p}^{2}$ < 0.001; BF_10_ = 0.07]. Hence, subjects had similar guilt ratings when the scenario was presented in DSF language (*M* = 3.34, *SD* = 1.93), and non-DSF language (*M* = 3.28, *SD* = 2.01). The interaction was also not significant [*F*(2,1891) = 1.537, *p* = 0.2.15, $\eta_{p}^{2}$ = 0.002; BF_10_ = 0.054]. Bayesian analysis suggested there is strong evidence that DSF plays no role in the attribution of guilt. Further, it suggested that the model with the largest posterior probability given the data was the model including only the culpability factor, with P(model| data) = 0.94. *Post hoc* analysis revealed that the data and conclusions remained qualitatively the same when not enforcing any of the exclusion criteria. Therefore, it is unlikely that these results are due to the high exclusion rate.

One potential explanation for the above results is the following. It might be that the DSF language did drive subjects to ascribe less guilt to the defendant. However, at the same time, the following occurred. The DSF language nudged subjects to more dualistic views, and those more dualistic view pushed subjects to attribute more guilt to the defendant. Hence, these two processes countered each other, resulting in no effect of DSF language on guilt attribution. To test this, we carried out additional *post hoc* analyses on the influence of subjects’ tendency toward dualistic views on their attribution of guilt. Subjects were overall non-dualistic, with only 9% reporting dualistic viewpoints for each of the following questions: (1) Do you believe that an individual and their brain are separate entities? (2) Do you believe that an individual and their mind are separate entities? (3) Do you believe that an individual’s mind and brain are separate entities? Further, focusing on subjects who reported either strong dualistic or non-dualistic views (6–7 or 0–1 for question 1 above, respectively), we found no dependence of the reported dualistic views on DSF language. An ANOVA with DSF and culpability as independent factors and dualistic views as the dependent factor revealed no effect for DSF on dualistic views [*F*(1,1266) = 0.144, *p* = 0.71, $\eta_{p}^{2}$ < 0.001]. (Running similar ANOVA analyses on strong dualistic and non-dualistic views according to questions 2 and 3 resulted in no significant differences either).

To test whether dualistic views themselves influenced the attribution of guilt, we performed Bayesian ANOVAs with the factors culpability, DSF language, and dualistic views. Model comparisons identified the best model (and whether it included the factor of dualistic views), and whether this model was better than the other models (that is, the other models had BF_10_< 0.33). The first question about dualistic views did not appear to relate to how people judged guilt. The best model included only culpability as a factor [P(M| data) = 0.76], and the next strongest model, which included also the factor of dualistic views, had a BF_10_ of 0.14 against the best model. The second question, on the other hand, did appear to be related to how people judge guilt: the best model included the main factors of culpability, and dualistic views, as well as an interaction between these two factors [P(M| data) = 0.60]. The next best model included only culpability as a factor, and had a BF_10_ of 0.28 against the best model. Both the main effect of dualistic views, as well as the interaction appear to be mainly driven by an increase in perceived guilt in the uncertain scenario in the group with dualistic views. Critically, however, this did not have any bearing on the (null) effect of DSF. The third question yielded results similar to the second question. The best model included the main factors of culpability, and dualistic views, as well as an interaction between these two factors [P(M| data) = 0.64]. The next best model included the main factors of culpability and dualistic views, and had a BF_10_ of 0.32 against the best model. Again, both the main effect of dualistic views, as well as the interaction appear to be mainly driven by an increase in perceived guilt in the uncertain scenario in the group with dualistic views. Thus, it seems like a more dualistic view is related to stricter guilt judgments, when guilt (or its absence) is not clearly defined by the text.

### Discussion

As opposed to Experiment 1, Experiment 2 revealed no evidence for the effect of DSF language on the assignment of guilt for intent to kill—the effect was both negligible and not significant. This null result was further corroborated by the Bayesian analysis, which yielded strong evidence that DSF language has no effect on the assignment of guilt for intent to kill. This mitigates the concern that the study was not powerful enough to detect the effect. In addition, the clear and strong effect of culpability on assigning guilt for intent to kill, provides evidence against explaining the results via some type of non-compliance of our subjects with our instructions.

An interesting additional finding, however, was that a person’s dualistic views may influence their assignment of guilt for intent to kill, in ambiguous scenarios. Individuals who were more prone to dualistic views perceived agents as guiltier in the uncertain scenario. This could potentially provide an explanation to the lack of DSF effect: Arguably, DSF language might have a twofold effect. On the one hand, it makes the agent appear less guilty (as this was ‘his brain’s fault’). But, on the other, it reinforces dualistic intuitions, which appear to elevate guilt judgments. Critically, however, the latter part of this potential explanation was not borne out by our data, as we found no effect of DSF on dualistic intuitions. Yet, again, this might reflect the insensitivity of our measures for assessing dualistic intuitions here. Thus, future studies are needed to better understand the relations between dualistic thought, assigned guilt, and, potentially, the DSF.

Two alternative explanations for the lack of DSF should be considered, however. One is that the results of Experiment 1 were a false-positive outcome. Another is that the DSF language was less effective for this specific scenario, for some reason yet to be determined. To decide between these two opposing interpretations, we conducted Experiment 3.

## Experiment 3

Given the conflicting results of Experiments 1 and 2, Experiment 3 was run as a direct replication of Experiment 1, again focusing on perceived guilt. If the results of Experiment 1 proved to be reproducible, the claim that DSF language might affect judgments of guilt would be strengthened, though limited to the specific moral scenario spelled out in Experiment 1. Alternatively, if again an effect was not found, it would indicate that—contrary to our theoretical suggestion (Mudrik and Maoz, 2014) and hypothesis—DSF language might not affect the subjects’ assignment of guilt to defendants. Similar to Experiment 2, this experiment—including sample size, methods, hypotheses and analysis plan—was preregistered in the OSF^3^.

### Methods

#### Subjects

The same sample size as in Experiment 2 was pre-defined (minimum 1,794 subjects; 1,810 were collected of which 955 were females; aged 18–80, mean ± std age = 37.5 ± 12.3 years old). All subjects were again gathered using MTurk and Qualtrics, with the experiment open only to subjects who had not previously participated in Experiments 1, 2 or in piloting. As this was a critical replication study, we wanted to make sure that our data was as clean as possible and that it included only subjects with histories of valid survey completion. Hence, we opened the study only to MTurk users that had previously completed 1,000 or more surveys on MTurk with a 95% or higher HIT approval rating. We further modified the exclusion criteria to avoid the strikingly high exclusion rate in Experiment 2. Thus, we kept only criteria (a) and (b) above (rating the defendant relatively innocent or guilty in the Guilty or Innocent condition, respectively). Subjects who provided a Worker ID that is different than their actual MTurk Worker ID (61 excluded subjects) and subjects who provided an invalid completion code or if the code they provide does not match the code assigned at survey completion (11 excluded subjects) were also excluded. We added an additional attention check where subjects rated how *not guilty* the defendant was. Then, subjects who rated the defendant as a 1–2 on the 7-point Likert scale for guilt and also rated the defendant as a 1–2 on the 7-point Likert scale for no guilt, or conversely rated the defendant as a 6–7 on the 7-point Likert scale for guilt and also rated the defendant as a 6–7 on the 7-point Likert scale for no guilt were excluded (176 subjects) as these answers were interpreted as inconsistent. Overall, we collected 2,259 subjects, out of which 449 were excluded, yielding a final sample of 1,810 subjects.

#### Design, Apparatus, Stimuli, and Procedure

The moral scenario was identical to that used in Experiment 1. However, several modifications were introduced. As previously described, we added a question opposing the original measure of perceived defendant guilt to test for consistency. Thus, the moral scenario was followed by the following questions: (a) “How much do you agree or disagree with the following sentence: ‘Jim is guilty of the charge of vehicular assault with intent to kill”’ (1 – “strongly disagree” to 7 – “strongly agree”; Experiment 1) and (b) “How much do you agree or disagree with the following sentence: ‘Jim is not guilty of the charge of vehicular assault with intent to kill”’ (1 – “strongly disagree” to 7 – “strongly agree”). This was used to exclude inconsistent subjects (see above).

Similar to Experiment 2, an Additional Measures block followed the moral scenario where subjects were asked to rate (a) if they were confident in their decision, (b) if they regret their decision, and (c) if they would like to change their decision on 7-point Likert scales (1 – “no” to 7 – “yes”).

### Results

**Figure 3:**
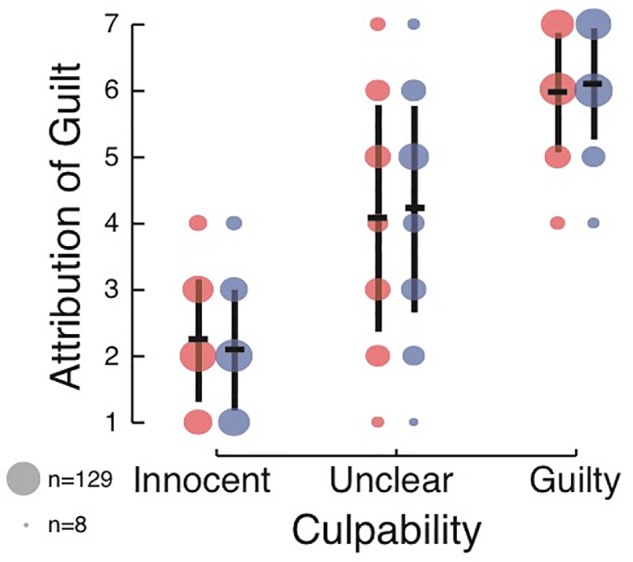
Effect of culpability and DSF language on rating of intent to kill in Experiment 3. Annotation is the same as that used in Figure 1, 2.

A two-way ANOVA with culpability and DSF revealed a main effect of culpability [*F*(2,1804) = 1584.28, *p* < 0.001, $\eta_{p}^{2}$ = 0.637, BF_10_ = 2.1 × 10^393^, see Figure 3]. Similar to Experiment 1 and Experiment 2, subjects had the highest ratings of defendant guilt in the Guilty condition (*M* = 6.04, *SD* = 0.87), followed by the Unclear condition (*M* = 4.15, *SD* = 1.63) and then the Innocent condition (*M* = 2.17, *SD* = 0.92). Yet—as opposed to the results of Experiment 1, and in line with those of Experiment 2—no effect of DSF language was found [*F*(1,1804) = 0.563, *p* = 0.45, $\eta_{p}^{2}$ < 0.001, BF_10_ of 0.067], with similar judgments of defendant guilt in the DSF language (*M* = 4.09, *SD* = 1.96) and non-DSF language (*M* = 4.15, *SD* = 2.00) conditions. The BF suggested that the null model was about 15 times more likely than the alternative model, in which DSF would have an effect therefore suggesting that the DSF did not have an effect on subjects’ ratings of defendant guilt. The interaction was trending, although non-significant, using traditional null-hypothesis statistical testing [*F*(2,1804) = 2.75, *p* = 0.064, $\eta_{p}^{2}$ = 0.003]. However, a Bayesian analysis suggested moderate evidence for the absence of an interaction (BF_10_ = 0.18). Overall, the model with the largest posterior probability given the data was the model including only the culpability factor, with P(model| data) = 0.93. *Post hoc* analysis again revealed that the data and conclusions remained qualitatively the same when not enforcing any of the exclusion criteria.

We further checked whether the covariates age and gender influenced the results. An ANCOVA^4^ revealed an effect of culpability [*F*(2,1802) = 1576.79, *p* < 0.001, $\eta_{p}^{2}$ = 0.636], no effect of DSF [*F*(1,1802) = 0.584, *p* < 0.44, $\eta_{p}^{2}$ < 0.001], and no interaction [*F*(2,1802) = 2.76, *p* < 0.063, $\eta_{p}^{2}$ = 0.003] on subjects’ ratings of defendant guilt. Covariate effects were also non-significant [sex: *F*(1,1802) = 0.459, *p* = 0.498, $\eta_{p}^{2}$ < 0.001; age: *F*(1,1802) = 0.794, *p* = 0.373, $\eta_{p}^{2}$ < 0.001]. These results are consistent with the ANOVA analysis reported above.

We also tested whether the effect existed only for some subgroups of our subjects and was then washed out over the entire population. This could have happened either because the effect of DSF language was limited to a small subgroup, or because the DSF language had opposite effects across different subgroups and was then canceled out when they were combined. In particular, we tested whether age or gender affect the DSF using a linear regression. In other words, we regressed the attribution of guilt on: DSF, age, gender, their pairwise interactions, and their triple interaction. The overall regression reflected no significant trends [*F*(7,1802) = 1.055, *p* = 0.39, *R^2^* = 0.004]. Neither did any of the variables exhibit significant trends (|*t*| < 1.65 and *p* > 0.1 for all variables except the intercept). We therefore conclude that demographic differences could not have explained our null result.

Finally, in this experiment the questions related to regret, confidence, and the desire to change the decision were not used as basis for subject exclusion. We therefore analyzed here (*post hoc*) whether there were systematic differences in the responses to these questions that were dependent on one of the factors in the experiment. We found that confidence was affected by culpability [*F*(2,1804) = 40.75, *p* < 0.001, $\eta_{p}^{2}$ = 0.043; BF_10_ = 8.1 × 10^14^], so that subjects were less confident (*M* = 5.13, *SD* = 1.42) in the unclear condition than in the guilty (*M* = 5.80, *SD* = 1.18) or the innocent (*M* = 5.58, *SD* = 1.33) conditions. There was no effect of DSF language [*F*(1,1804) = 0.049, *p* = 0.83, $\eta_{p}^{2}$ < 0.001; BF_10_ = 0.054], nor an interaction [*F*(2,1804) = 1.22, *p* = 0.296, $\eta_{p}^{2}$ = 0.001; BF_10_ = 0.04].

Regret was also affected by culpability [*F*(2,1804) = 24.87, *p* < 0.001, $\eta_{p}^{2}$ = 0.027; BF_10_ = 1.85 × 10^14^], so that subjects reported more regret as guilt increased. There was no effect of DSF language [*F*(1,1804) = 0.085, *p* = 0.77, $\eta_{p}^{2}$ < 0.001; BF_10_ = 0.056], nor an interaction [*F*(2,1804) = 3.052, *p* = 0.048, $\eta_{p}^{2}$ = 0.003; BF_10_ = 0.24]. A similar pattern was found for the desire to change a decision, indexed by a main effect for culpability [*F*(2,1804) = 23.22, *p* < 0.001, $\eta_{p}^{2}$ = 0.025; BF_10_ = 3.96 × 10^7^]. There was no effect of DSF language [*F*(1,1804) = 0.084, *p* = 0.83, $\eta_{p}^{2}$ < 0.001; BF_10_ = 0.054]. There is a an interaction [*F*(2,1804) = 3.052, *p* = 0.048, $\eta_{p}^{2}$ = 0.002; BF_10_ = 0.077], though it is not supported by the Bayesian analysis (and would not have survived correction for multiple comparison).

### Combining Data From All Experiments

Combining data from all three experiments, a two-way ANOVA with culpability and DSF as factors revealed a main effect of culpability [*F*(2,4310) = 2683.79, *p* < 0.001, $\eta_{p}^{2}$ = 0.555, BF_10_ = 5.97 × 10^752^]. The DSF factor was non-significant and a Bayesian analysis provided moderate evidence against this effect [*F*(1,4310) = 3.31, *p* < 0.069, $\eta_{p}^{2}$ = 0.001, BF_10_ = 0.146]. An interaction was also not found [*F*(2,4310) = 2.00, *p* < 0.135, $\eta_{p}^{2}$ = 0.001, BF_10_ = 0.04].

### Discussion

The results of Experiment 3 align with those of Experiment 2; no effect for DSF language was found. Even when collapsing the results of all three experiments together (now including several thousands of subjects), no effect was found, and substantial (though moderate) evidence against the effect was obtained. In the general discussion below, we suggest an explanation to what appears to be a lack of effect of DSF phrasing on subjects’ ratings.

## General Discussion

In this study, we ran three experiments to test whether phrasing some facts about the actions of agents using the Double-Subject Fallacy (DSF; e.g., “Bob’s brain makes him have aggressive feelings more often than most people”) results in these agents being perceived as less guilty, compared to when the same facts are not phrased using the DSF (e.g., “An area of Bob’s brain, commonly associated with aggression, is more active,” respectively). In the first, exploratory, experiment we tested whether the DSF has an effect on the perception of guilt in a hypothetical case of vehicular assault. We created a moral scenario, manipulated to represent increasing defendant culpability in a court case. Conditions were designed to present the defendant as guilty, ambiguous, or as innocent. Each culpability condition also included neuroscientific evidence, either using DSF language or non-DSF language (in a between-subjects design). We found a clear effect of culpability on both subjects’ ratings of defendant guilt and on their recommended jail sentences, validating our manipulation and confirming that subjects included in the experiments were reading the scenarios and following instructions. We also found a significant, but weak, effect of DSF language on subjects’ ratings of defendant guilt. However, there was no effect of DSF language on subjects’ recommended jail sentences.

To test the replicability of this effect, and its generalizability to other moral scenarios, we ran Experiment 2 (following a power analysis and preregistration). There, we tested whether the DSF has an effect on the perception of guilt in another moral scenario involving bodily harm using the same experimental design. We again found the expected effect of culpability but failed to find an effect of the DSF language on the perception of guilt. To understand these conflicting results, we ran Experiment 3 as a simple replication of Experiment 1. We once more found a strong effect of culpability, but not of DSF. This null result was further supported by a Bayesian analysis that we conducted on all three experiments collapsed together, which yielded even more substantial evidence against the effect. The most straightforward interpretation of our results is that DSF language has no effect on defendant guilt assessment, and therefore moral responsibility, at least for the moral scenarios tested.

Might it nevertheless be that the effect exists, yet we failed to find it? Based on the power of this study, this appears unlikely. The effect was only found in Experiment 1, which was exploratory. Experiments 2 and 3 were designed following a power analyses and both had power of 0.88, and sampled 1,794 and 1,897 subjects, respectively. Thus, the probability that we would fail to capture the effects, if they were present, were 0.12 in each. As the two experiments were conducted on disjoint subjects, computers, and times using different moral scenarios, the joint probability that both experiments failed to capture the effect when it was present is likely much lower, perhaps as low as 0.12 × 0.12 = 0.014. Therefore, our study is unlikely to be statistically underpowered.

Yet the DSF manipulation itself might be underpowered, of course. And so, it could be that the differences between the DSF and non-DSF language where too subtle, and accordingly – did not evoke an effect. In an effort to make the conditions comparable, sentence structure and length were held similar in this study. But in courtrooms, arguments along the lines of “my brain made me do it” (Morse, 2004; Gazzaniga, 2006; Sternberg, 2010; Maoz and Yaffe, 2015) could and would probably be made much more explicitly. Thus, it is possible that the non-DSF language may still show effects in legal situations, even though we did not find it here.

Finally, another option is that subjects found the DSF language more confusing than non-DSF language, which might influence their guilt ratings. Indeed, we previously claimed that DSF language might not only preserve dualistic intuitions, but also evoke confusion (Mudrik and Maoz, 2014). If this was the case, this could have been reflected in the exclusion of more subjects under the DSF than under the non-DSF condition. Indeed, when comparing the exclusion percentages between the two conditions, DSF tended to be excluded slightly more than non-DSF: not confident of response, 7 vs. 8%; not feeling responsible for outcome, 40.33 vs. 39%; not understand Guilty condition, 25 vs. 25%; not understand innocent condition, 13 vs. 16%; regrets decision, 9 vs. 11.67%; wishes to change decision, 7 vs. 9.67%; and legal knowledge, 31.67 vs. 33.33%; for DSF vs. non-DSF, respectively. However, none of these comparisons was statistically significant (binomial test, *p* > 0.31 for all conditions).

How are we to understand our results then? The results seem quite conclusive: in the scenarios we presented, DSF language had no impact on subjects’ assignments of guilt. The results of the first experiment, accordingly, seem to be an example of a false-positive. Of course, we should not over-interpret null results, and it could still be that we failed to find the effect due to some problem with our methods or design. Thus, we conclude this paper by acknowledging that currently there is no evidence that DSF language affects actual judgments of guilt. However, this does not render our other claims against DSF (see again Mudrik and Maoz, 2014) invalid; conceptually speaking, it is still a confused form of writing, and—perhaps more importantly—it is dualistic in nature. Thus, we still argue that to promote a clearer understanding of the intricate relations between the brain and the conscious self, scientists should avoid DSF writing. Further studies should empirically examine the potential influences of such DSF writing on subjects’ comprehension and on their dualistic intuitions.

A final, unrelated, point about our study has to do with the proper interpretation of preregistration studies, especially given the widely discussed ‘replication crisis,’ both within and outside cognitive neuroscience and psychology (Nosek et al., 2015; Munafò et al., 2017). We initially discussed whether to preregister the pilot study we carried out before starting it. Had we done so, instead of treating it as just a pilot study for future replications, we might have concluded that our results bore out our hypothesis, that DSF language makes people appear less guilty. Thus, preregistration by itself is not enough to reduce the chance of obtaining false-positive results and reaching erroneous conclusions. Two further things are worth mentioning about this point. One is that preregistration should come together with a power analysis, as we did in Experiments 2 and 3 here. There we know what the probability is of false-positive and false-negative results. The second is almost trivial, though important: preregistration is no substitute for replication (Koole and Lakens, 2012; Nosek et al., 2012). An additional methodological note refers to Bayesian analysis as compared with traditional Null Hypothesis Statistical Testing; while the latter indicated a significant result of the DSF manipulation in Experiment 1, the former implied that this result is inconclusive, and if anything – suggested that the data is more consistent with a lack of effect (that is, the null result model was twice as likely than the effect model). As the result of Experiments 2 and 3 indeed suggest that an effect does not exist, this study demonstrates the importance of Bayesian statistics in assessing the obtained effects, or lack thereof (Gelman et al., 2014; Kruschke, 2014; Vandekerckhove et al., 2018; Wagenmakers et al., 2018).

## Ethics Statement

This study was carried out in accordance with the recommendations of IRB#15-001094, UCLA IRB North with written informed consent from all subjects. All subjects gave written informed consent in accordance with the Declaration of Helsinki. The protocol was approved by UCLA IRB North.

## Author Contributions

UM, JvB, and LM conceived the study and wrote the manuscript. KS and UM ran the experiments and analyzed the results. JvB reanalyzed the results. UM, KS, JvB, and LM commented on the manuscript and revised it.

## Conflict of Interest Statement

The authors declare that the research was conducted in the absence of any commercial or financial relationships that could be construed as a potential conflict of interest.

## Footnotes

1 https://osf.io/wj6my

2 It turns out that we made a mistake when computing the required sample size that would achieve power of 0.95 power. In fact, 1,794 subjects achieve only power of 0.88. Though lower than we initially intended, this is still a reasonable power. Moreover, the overall analysis that we carried out (see below), incorporating subjects from all experiments, yielded the same results, suggesting that our conclusions do not stem from lack of power.

3 https://osf.io/a8cq2

4 First, we tested whether the covariates were independent of the treatment effects to ensure we could perform an ANCOVA. To that end, we performed ANOVAs with factors culpability and DSF, and dependent variables age and gender. There were significant effects of culpability on age [*F*(2,1804) = 3.591, *p* = 0.028, $\eta_{p}^{2}$ = 0.004], and DSF on gender [*F*(1,1804) = 4.49, *p* = 0.034, $\eta_{p}^{2}$ = 0.002], suggesting that covariates were not independent of the treatment effects. However, due to the very small effect sizes, we decided to continue performing the ANCOVA.
